# The clinical manifestations and pregnancy outcomes of COVID-19 infection at a tertiary care hospital

**DOI:** 10.12669/pjms.40.2(ICON).8949

**Published:** 2024-01

**Authors:** Aisha Syed Wali, Maria Mushtaq Ali, Rabia Bibi, Anum Rahim

**Affiliations:** 1 Aisha Syed Wali, Consultant, Obstetrics & Gynecology Department, Sheikh Saeed Memorial Hospital (SSMH), The Indus Hospital and Health Network, Karachi, Pakistan; 2 Maria Mushtaq Ali Office of the Research, Innovation and Commercialization (ORIC). The Indus Hospital and Health Network, Karachi, Pakistan; 3 Rabia Bibi Obstetrics & Gynecology Department, Sheikh Saeed Memorial Hospital (SSMH), The Indus Hospital and Health Network, Karachi, Pakistan; 4 Anum Rahim Department of Community Health Sciences, Agha Khan University, Karachi, Pakistan

**Keywords:** COVID-19 infection, Pregnancy, Clinical manifestations, Pregnancy Outcome

## Abstract

**Objective:**

To evaluate clinical presentation and pregnancy outcomes in pregnant women with Covid-19 infection in our local tertiary care from lower middle-income country.

**Methods:**

A retrospective study was conducted at Obstetrics & Gynecology department, Sheikh Saeed Memorial Hospital (SSMH) of The Indus Hospital and Health Network (IHHN) from March 2020 to August 2021. Data of 422 admitted pregnant women with COVID-19 infection was retrieved for demographic and clinical information, laboratory tests, pregnancy outcome, and neonatal outcomes on RED-Cap and analyzed on SPSS 26. Univariate and multivariable logistic regression analyses were performed to estimate odds ratios (OR) for symptomology with categorical variables and feto-maternal outcome.

**Results:**

Of the total 422 pregnant women, 24.4% were symptomatic, 74.7% exhibiting mild symptoms. Largely reported symptoms were fever (71.8%), cough (36.9%) and body ache (35.0%); while odds of symptomatic COVID-19 infection was less in educated pregnant women (OR 0.3; 95% CI 0.1-0.9) compared to uneducated. Amongst maternal comorbidities, odds of having symptomatic COVID-19 infection were 3.8 times (95% CI 1.1-13.0) in women with chronic hypertension and 5.5 times (95% CI 2.9-10.4) in women with diabetes. Symptomatic women had significantly greater incidence of miscarriages (p= 0.009), PPROM (p= 0.001), preterm birth (p= 0.000), preeclampsia (p= 0.000), placental abruption (p= 0.006) and maternal ICU admission (p= 0.000) than asymptomatic patients. Still birth was higher (6.4% vs 1.3%, p-value= 0.013) in symptomatic group. The odds of having severe maternal outcome were higher (OR=3.5; 95% CI 1.9-6.0) in symptomatic pregnant women.

**Conclusion:**

Majority of pregnant women were asymptomatic. Symptomatic women with COVID-19 infection had an increased risk of adverse feto-maternal outcome.

## INTRODUCTION

COVID-19 infection was a major burden in the world sometime back and remains a threat with emerging new variants. As of May 2023, 765,903,278 confirmed cases of COVID-19 infection have been reported, including 6,927,378 deaths.^1^ The knowledge gained from previous human coronavirus outbreaks suggests that pregnant women and their fetuses are particularly susceptible to poor outcomes. Physiological changes in pregnancy including immunosuppression, make pregnant women more vulnerable to respiratory pathogens and acute pneumonia due to intolerance to hypoxia.^2^ In June 2020, the Centers for Disease Control and Prevention (CDC) reported that hospitalization with COVID-19 infection occurred in 31.5% of pregnant women compared with 5.8% of nonpregnant women.^3^

Although understanding of this disease is growing every day, many queries regarding diagnosis, clinical management, the impact of the disease on pregnant women and newborns, and the potential of mother-to-child transmission are still being explored. There are currently few studies examining the prevalence of COVID-19 infection in the obstetric community and its effects on perinatal outcomes. Moreover, there is a gap in the evidence because majority of published studies are case reports or series written in Chinese language. Quantification of the rates of COVID-19 infection, its risk factors, clinical manifestations and outcomes are key to planning maternal care and management after an evolving pandemic scenario. Cases are still being reported in pregnancy with risk of adverse outcomes.^4^

The aim of this study was to evaluate the clinical presentation and pregnancy outcomes in pregnant women who presented in our local setting with COVID-19 infection.

## METHODS

In this retrospective study inpatient data from hospital records of 422 pregnant women diagnosed with COVID-19 infection, admitted at OBGYN of SSMH of IHHN who either delivered or miscarried from March 2020 to August 2021, was analyzed retrospectively. Clinical characteristics, laboratory test results, pregnancy outcomes were collected from the Hospital’s Medical Information System and entered on RED-Cap. Adverse maternal outcomes were miscarriages, preterm labor, premature rupture of membranes (PPROM), preterm birth, preeclampsia, placental abruption and maternal ICU admission.

### Ethical Approval

The study was approved by IHHN’s IRB (IHHN, IRB# IRD_IRB_2020_08_01).

### Inclusion criteria

All PCR-positive pregnant women admitted at SSMH of IHHN during study period.

### Exclusion criteria

PCR-positive pregnant women referred out before admission.

### Statistical Analysis

Pregnant women with COVID-19 infection were categorized into two groups based on symptomatology. Data was analyzed via SPSS 26. Median (IQR) were reported for quantitative variables such as age, laboratory parameters, etc. whereas frequency and percentages were calculated for qualitative variables such as mode of admission, symptoms, comorbidities, maternal outcomes, parity, etc. Odds ratios of exposures with 95% CIs were calculated for the demographics, clinical characteristics, duration of hospital stay, and adverse maternal outcome to compared symptomatic and asymptomatic groups.

Multivariate analysis was done in order to find an optimal model where all the included covariates maintained a significant association with symptomology. In multivariate analysis backward selection method was applied on all factors that were significant (≤ 0.25) in the univariate analysis. Following the discovery of a model with only significant (≤ 0.05) covariates, each omitted covariate was gradually brought back into the model and kept if significant. Chi-square / fisher exact test was applied for association of qualitative variable including status of birth, mode of delivery, feto-maternal outcomes and laboratory findings with the symptomology. P-value of 0.05 was considered significant.

## RESULTS

Form a total of 5349 women who delivered during the study duration, 422 (7.89%) were COVID-19 positive, and included in the study. Median age of patient was 27 (IQR=23-30) years and median gestational age at time of delivery was 37 (IQR=34-39) weeks. One fourth of the women were symptomatic (24.4% n = 103/422). Characteristics of both groups are presented in (Table-I).

**Table-I:** Comparison of sociodemographic characteristics and comorbid conditions

Independent Variable	Symptomatic N (%) 103 (24.4)	Asymptomatic N (%) 319 (75.6)	P- value	Non-adjusted OR (95%CI)	P- value	Adjusted OR (95%CI)
Age in years median (IQR)	27(23-30)	27(23-30)	-	-	-	-
<35 years	92 (89.3)	286 (89.7)	Ref	-	-	-
≥35 years	11 (10.7)	33 (10.3)	0.923	1.0(0.5-2.1)	-	-
***Education***						
Uneducated	41(39.8)	61(19.1)	Ref	-	-	-
Primary	18(17.5)	47(14.7)	0.101	0.5(0.3-1.1)	0.165	0.6(0.3-1.2)
Secondary	38(36.9)	187(58.6)	0.000	0.3(0.2-0.5)	0.000	0.3(0.1-0.6)
Graduate or above	6(5.8)	24(7.5)	0.048	0.4(0.1-1.0)	0.060	0.3(0.1-1.0)
***Parity***						
Primiparity	25 (24.3)	104 (32.6)	Ref	-	-	-
Multiparity	78 (75.7)	215 (67.4)	0.261	1.5(0.9-2.5)	-	-
***Comorbid Conditions***						
***Chronic Hypertension***						
No	97(94.2)	314 (98.4)	Ref	-	-	-
Yes	6 (5.8)	5 (1.6)	0.028*	3.8(1.1-13.0)	-	-
***Diabetes Mellitus***						
No	75 (72.8)	299 (93.7)	Ref	-	-	-
Yes	28 (27.2)	20(6.3)	0.000*	5.5 (2.9-10.4)	0.000*	5.0(2.6-9.5)
***Gestational Diabetes***						
No	71 (68.9)	235 (73.7)	Ref	-	-	-
Yes	32 (31.1)	84 (26.3)	0.350	1.2(0.8-2.1)	-	-

OR, odds ratio; CI, confidence interval,

* = Statistically significant with p value ≤ 0.05.

The odds of having symptomatic COVID-19 infection was found to be less in educated pregnant women (OR 0.3; 95% CI 0.1-0.9). No significant difference existed in maternal age and parity in both study groups. With maternal comorbidities, odds of having symptomatic COVID-19 infection was 3.8 times more (95% CI 1.1-13.0) in women with chronic hypertension in univariate analysis. Similarly, pregnant women with diabetes mellitus had greater chances of symptomatic COVID-19 infection, 5.5 (95% CI 2.9-10.4) as compared to non-diabetic women. In multivariate analysis years of education and diabetes remained independently associated with symptomatic COVID-19 infection (Table-I).

Anemia was significantly more prevalent in symptomatic population however, lymphocytopenia was significantly predominant in asymptomatic patients in contrast to symptomatic group. Significant difference (p-value= 0.013) was observed in birth outcomes with higher prevalence (6.4%) of still birth in symptomatic group verses asymptomatic group (1.3%). (Table-II)

**Table-II:** Comparison of laboratory findings in symptomatic and asymptomatic patients

Independent Variable	Symptomatic N (%) 103 (24.4)	Asymptomatic N(%) 319 (75.6)	Total N(%) 422	P-value
***Hemoglobin gm/dl***				0.000*
Median Hb	10 (9-12)	11(10-12)	11(10-12)	
Anemic (Hb<10.5) N (%)	58^b^ (56.3)	104 (32.6)	162 (39.4)	
Normal N (%)	45 (43.7)	215^a^ (67.4)	260 (61.6)	
***Platelet count x10^9^/L***				0.716
Median platelet counts (IQR)	210 (136-270)	230 (179-281)	222(174-280)	
Thrombocytopenia N (%)	28 (27.2)	47 (14.7)	75 (17.8)	
Normal N (%)	71 (68.9)	261 (81.8)	332 (78.7)	
Thrombocytosis N (%)	4 (3.9)	11(3.4)	15 (3.5)	
***Leukocyte count x10^9^/L***				0.176
Median leukocyte count	12 (7.7-17)	10 (8.2-12)	104 (8-12.6)	
Leukopenia N (%)	1 (1.0)	5 (1.6)	6 (1.4)	
Normal N (%)	43 (41.7)	164 (51.4)	207 (49.1)	
Leukocytosis N (%)	59 (57.3)	150 (47.0)	209 (49.5)	
***Lymphocyte Count %***				0.000 *
Median Lymphocyte count	23 (21.0-27.0)	20 (16-25)	22 (17-25)	
Lymphocytopenia N (%)	21 (20.4)	140 ^a^ (43.9)	161 (38.2)	
Normal N (%)	79 ^b^ (76.7)	177 (55.5)	256 (60.7)	
Lymphocytosis N (%)	3 (2.9)	2 (0.6)	6 (1.2)	

Chi square test was applied,

* = Statistically significant with P value ≤ 0.05.

In multiple regression analysis after adjusting for age, education status, diabetes, maternal outcomes, and mode of delivery, odds for prolonged hospital stay were higher in symptomatic COVID-19 infection (OR 5.5; 95% CI 2.9-10.5), Table-III.

**Table-III:** Comparison of hospital stay in symptomatic and asymptomatic groups.

	Median days	Normal stay N (%)	Prolonged Stay N (%)	P -value	Non-Adjusted OR (95% CI)	P-value	Adjusted OR (95% CI)
Symptomatic	3 (2-5)	25 (24.3)	78 (75.7)	0.000*	5.6 (3.4-9.2)	0.000*	5.5 (2.9-10.5)
Asymptomatic	2 (1-2)	205 (64.3)	114 (35.7)				

OR = odds ratio; CI = confidence interval;

* = Statistically significant.

We further categorized the symptomatic group into mild and moderate-severe symptoms based on severity of symptoms. Of 103 symptomatic women with COVID-19, 77 women (74.7%) had mild symptoms. The most reported symptoms were Fever (71.8%), cough (36.9%) and body ache (35.0%). Adverse maternal outcomes were observed in 89 (21.1%) pregnant women (Table-IV).

**Table-IV:** Overall comparison of adverse maternal outcome in symptomatic and asymptomatic groups.

	Adverse Maternal outcome N (%)		P-value	Non-adjusted OR (CI 95%)	P-value	Adjusted OR (CI 95%)

	Yes	No				
Symptomatic (103)	48 (46.6)	55 (53.4)	0.000*	5.9(3.6-9.7)	0.000*	3.1(1.8-5.4)
Asymptomatic (319)	41 (12.9)	278 (87.1)				

OR, odds ratio; CI, confidence interval;

* = Statistically significant.

**Fig.1:**
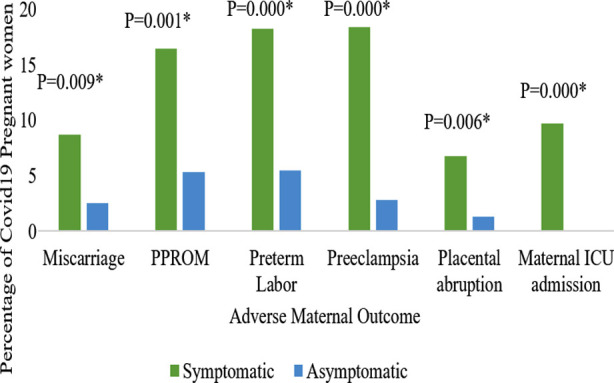
Comparison of maternal outcomes in study groups Chi square test was applied, * = Statistically significant with P value ≤ 0.05 ***Note:*** During frequency and Chi-Square application of preterm labor, 17 cases of miscarriage were excluded.

Symptomatic women had significantly greater incidence of miscarriages, PPROM, preterm birth, preeclampsia, placental abruption and maternal ICU admission than asymptomatic patients, among which preterm labor was predominant (Figure 1). Symptomatic COVID-19 infection was found to be an independent predictor of severe maternal outcomes in multivariate analysis with higher Odds (OR=3.1; 95% CI 1.8-5.4) after controlling all other confounding variables including maternal age, years of education, chronic hypertension, diabetes mellitus and duration of stay.Regarding mode of delivery, non-significant difference (p-value = 0.064), with more cases of instrumental deliveries, was found in the symptomatic group (9.6%) compared to asymptomatic group (4.2%). However, no difference was observed between the groups in spontaneous vaginal (46.8% vs 56.8%) and cesarean deliveries (43.6% vs 39.0%).

## DISCUSSION

In the present study majority of the pregnant women were found to be asymptomatic similar to other national and international studies where majority of the COVID-19 patients, pregnant and general population, were asymptomatic or presented with mild respiratory symptoms.^5^-^7^ Fever, cough, and body ache were most common symptoms, 69.2%, 35.5%, and 34.6%, respectively. Similar to our results, local studies^8^,^9^ and a study by Chen et al. also identified fever and cough as its predominant clinical manifestations,^10^ whereas sore throat, chest pain, loss of taste and smell, diarrhea and difficulty in breathing were few other symptoms reported in these study. This spectrum of symptoms was similar to symptoms observed in non-pregnant population and in line with those of many previous studies on pregnant women.^10^-^12^

Regarding educational backgrounds a higher proportion of women in the symptomatic group lacked formal education compared to the asymptomatic group. Educated pregnant women had lower odds of experiencing symptomatic COVID-19 infections (OR 0.3; 95%; CI: 0.1-0.9). This aligns with findings from Lubeya et al, who also observed that women with a secondary level of education were less likely to contract COVID-19 compared to those with a primary level of education (OR: 0.23; 95% CI: 0.09–0.63).^13^

Previous research has shown that presence of other medical conditions alongside COVID-19 can lead to unfavorable results in pregnant women. In our study group, we found that pre-existing diabetes mellitus was the most common comorbid condition at 11.4%, followed by hypertension at 2.6%. This pattern aligns with what was reported by Huang et al.^14^ in their study, but it differs from a local study^8^ where hypertension was the most prevalent comorbidity. Similar association of hypertension was reported in a systematic review and meta-analysis, although in that study, there was no impact of diabetes in symptomatic women with COVID-19 infection.^15^ Our findings suggest that women with these comorbidities were more likely to experience symptomatic COVID-19 infections.

In our study, anemia (Hb<10.5g/dl) was significantly more common in symptomatic vs asymptomatic group (56.3% vs 32.6%, p-value 0.000). Smith et al in their prospective metanalysis also report association of anemia with increased risk of intensive care unit admission (OR, 1.63; 95% CI, 1.25-2.11). Low hemoglobin links to hypoxia, respiratory organ dysfunction, and therefore more adverse outcomes in COVID-19 infection.^16^ In consideration of lymphocyte count, it is noteworthy that majority (60.7%) of women, exhibited normal lymphocyte counts, contradictory to finding of a study by Khan et al.^7^, which reported a higher percentage (84%) of lymphocytopenia in pregnant women with COVID-19. A higher prevalence of lymphocytopenia was observed in the asymptomatic group compared to the symptomatic group (43.9% vs. 20.4%, with a p-value of 0.000), which is contradictory to the previous study where T lymphocytes and CD4+ T cell count below 200/ml revealed a significant relationship with symptomatology and mortality.^17^ In our study, mode of delivery had no statistical significance in either group. So far, there is lack of clear evidence regarding which delivery mode is better for COVID-19 cases. Many international guidelines recommended that the method and timing of delivery should be individualized, depending on the clinical status of the patients, gestational age, and the feto-maternal conditions.^18^,^19^

We found significant difference in adverse maternal outcomes among our study groups. In the symptomatic group, adverse maternal outcomes with significantly higher prevalence were, preterm labor and PPROM in contrast to observational data from Ireland and Denmark, that illustrated large declines in population-level rates of preterm birth during the COVID-19 pandemic, however, source of which is unknown.^20^ The above two adverse maternal outcome was followed by pre-eclampsia, similar to a study in which higher likelihood of pre-eclampsia was observed in women with symptomatic COVID-19 infection.^21^ The overall rate of maternal ICU admission rate was 2.4% in our symptomatic group comparatively very low (31%) than the data published by US National Notifiable Diseases Surveillance System.^3^ In an article summarizing findings of 108 pregnancies, COVID-19 infection during pregnancy was associated with maternal mortality^22^ but none observed in our study. However, data from a major referral hospital in East Java demonstrated a significantly higher risk of maternal death in COVID-19 infection cases.^23^ In different studies maternal mortality rate varied from 0-1.6% in pregnant women.^3^,^24^ In our study we observed significantly higher number of miscarriages in symptomatic pregnant women compared to asymptomatic; contradictory to a study conducted in Saudi Arabia, where rate of miscarriages did not significantly differ in symptomatic and asymptomatic Covid-19 pregnant women.^25^ In the present study no difference was discerned regarding maternal age, gestational diabetes, parity, leukocyte count and platelet count in symptomatic and asymptomatic groups.

Our study adds to currently available scanty data concerning COVID-19 infection in pregnant women in developing countries. This is a descriptive study with no selection bias and the gold standard tool PCR, was used for confirming COVID-19 infection.

### Limitations

This is a retrospective study; hence, we could not obtain additional hematologic and radiologic data to correlate with the severity of infection. Moreover, we studied women who had COVID-19 infection (7.89%) at the time of delivery or miscarriage, women who were COVID-19 positive at some point in pregnancy but became negative at time of delivery were not studied.

## CONCLUSION

The clinical spectrum of COVID-19 seen in pregnant women in our study was mostly asymptomatic or with mild symptoms only. Adverse maternal outcomes were significantly visible in symptomatic compared to asymptomatic pregnant women. A significant association was found between less educated, advanced age, and comorbid conditions like hypertension and DM with covid 19 in pregnant women.

### Authors’ contribution:

**ASW:** Conception, design and final approval of the version to be published.

**MMA:** Conception, analysis and interpretation of data; Drafting the article and revising it critically for important intellectual content; final approval of the version to be published.

**RB and AR:** Revising it critically for important intellectual content; and final approval of the version to be published. All authors agreed to be accountable for all aspects of the work in ensuring that questions related to the accuracy or integrity of any part of the work are appropriately investigated and resolved.
